# A Taste for Horseflesh: Dr. Killick Millard's Experience

**Published:** 1919-11-15

**Authors:** 


### A Taste for Horseflesh: Dr. Killick Millard's Experience.

Dr. C. Killick Millard, Medical Officer of Health for Leicester, in his annual report this year suggests that people should cultivate a taste for horseflesh. He says that during the early part of 1918, when there was a shortage of butcher's meat, three or four shops were opened for the sale of horseflesh in Leicester, and in two at least of them a considerable trade was done. The markets committee set aside a slaughter-house at the *abattoir* for the exclusive purpose of slaughtering horses, in order that the meat might be properly dressed.

Dr. Killick used horseflesh in his own household for a number of weeks, and no objection to it was raised either by family or servants. The chief difference in physical character between the flesh of the horse and that of the ox is that the former is darker and has a slightly sweet taste; also the fat does not set so firm when cold and is less palatable. But a lady in Leicester with a fairly large household informed Dr. Killick that she had been using it for some time without the family being aware of the fact. If a hospital cares to make the experiment no doubt the staff will volunteer to try this delicacy. When the staff has made the experiment some patients are sure to like to follow suit, and we await the result with interest.

